# Books

**Published:** 1883-09

**Authors:** 


### Books.

---

THE MICROSCOPE AND ITS REVELATIONS. By Wm. B. Carpenter, C.B., M.D., LL.D., F.R.S., F.G.S., F.L.S., etc. Sixth edition. Illustrated by twenty-six plates and five hundred wood engravings. Two volumes of about 350 pages each. New York: Wm. Wood & Co.

A proper notice of this valuable work would have appeared in our August number, but it was received just too late. And now that it is convenient to write it up, it would be as useless as out of place for us to attempt anything like a learned or elaborate review of such a work. On the contrary, it is quite sufficient and eminently modest just to say to our readers, the learned and the unlearned, the scientist and amateur, that Wm. Wood & Co. have just published in this country Dr. Carpenter's great work on the Microscope and Its Revelations—that it contains a great deal of abstruse, technical matter relating to the use of different angular apertures of objectiveness and their resolving power, and other like subjects which none but the scientific microscopist can understand or cares one straw about—that it contains in its lucid descriptions and explanations a vast deal of practical information for both the scientist and the student, and in its numerous plates and illustrations enough to delight, engage and instruct the amateur microscopist for a generation to come, or a life time.

In the field of microscopy, the ambitious student has much to encourage him; he has this great work of Dr. Carpenter's to direct his studies, and he has the abiding consciousness that the work will not be all done and things cleaned up before he can reach the scene of action.

He can rest assured that in the conquest of these interminable realms of infitesimal life, there will never be a microscopist Alexander blubbering over the lack of another world of the same sort to conquer.

---

Remarks on Hydrophobia. Read before the Phil. County Med. So., May, 1883. By Charles W. Dulles M.D., Surgical Registrar to the Hospital of the University, etc.

Dulles takes the ground that it is all bosh and non sense to talk about hydrophobia being a disease of any specific nature—a disease from the inoculation of a virus. It is only a disorder of the nervous system, caused by fear and trepidation, and none but people of that weakness can possibly fall into such an unmanly condition.

Now, our sagacity may be a duller thing than Dulles', but it does appear to us that such a specimen of agnosticism is exceedingly ridiculous under the facts.

How have little children, utterly unconscious of their danger, suffered and died, nevertheless, with all the symptoms of such a disease, and that too in every instance after having been bitten by a rabid dog? How can a dog, bitten by another dog known to be rabid, be said to take hydrophobia, merely from nervous dread of such a fate? Or, if these are not enough, how can a stolid, emotionless hog die of hydrophobia after being bitten by a rabid animal?

Would Dulles, under his theory, be willing—he should in the cause of science and humanity—to put his hand in the mouth of a mad dog? Bishop Berkley, the famous agnostic, taught something of the same sort of philosophy, when he asserted that what we see around us called matter is not a reality, but the mere image of things. Yet this same Bishop dodged a brick-bat thrown at his head all the same, and we rather opine that Dulles would give a snapping, rabid dog a wide wake, theory or no theory. A distinguished French physician recently assumed that inoculation from an indurated chancre would not impart syphilis. Like a man he tried it in his own arm, and promptly like a man he had a chancre, secondary syphilis, and now prostrated by the tertiary form of the disease

France is deprived of his able services. So much for beautifully spun theories.

